# Association between language barrier and inadequate prenatal care utilization among migrant women in the PreCARE prospective cohort study

**DOI:** 10.1093/eurpub/ckad078

**Published:** 2023-05-16

**Authors:** Maxime Eslier, Catherine Deneux-Tharaux, Thomas Schmitz, Dominique Luton, Laurent Mandelbrot, Candice Estellat, Rahmethnissah Radjack, Elie Azria

**Affiliations:** Université Paris Cité, CRESS, Obstetrical Perinatal and Pediatric Epidemiology Research Team, EPOPé, INSERM, INRA, Paris, France; Department of Obstetrics and Gynaecology, ELSAN—Polyclinique du Parc, Caen, France; Université Paris Cité, CRESS, Obstetrical Perinatal and Pediatric Epidemiology Research Team, EPOPé, INSERM, INRA, Paris, France; Department of Obstetrics and Gynaecology, Robert Debré Hospital, AP-HP, Paris Diderot University, Paris, France; Department of Obstetrics and Gynaecology, Beaujon-Bichat Hospital, AP-HP, Paris Diderot University, Paris, France; Department of Obstetrics and Gynaecology, Louis Mourier Hospital, AP-HP, Paris Diderot University, Colombes, France; Département de Santé Publique, Centre de Pharmacoépidémiologie (Cephepi), Sorbonne Université, INSERM, Institut Pierre Louis d’Epidémiologie et de Santé Publique, AP-HP, Hôpital Pitié Salpêtrière, CIC-1901, Paris, France; Maison des Adolescents—Youth Department, Paris University Hospital, University Hospital Cochin, Paris, France; University Paris-Saclay, UVSQ, Inserm, CESP, Team DevPsy, Villejuif, France; Université Paris Cité, CRESS, Obstetrical Perinatal and Pediatric Epidemiology Research Team, EPOPé, INSERM, INRA, Paris, France; Maternity Unit, Groupe Hospitalier Paris Saint Joseph, Paris, France

## Abstract

**Background:**

Inadequate prenatal care utilization (PCU) is involved in the higher risk of adverse maternal outcomes among migrant vs. native women. Language barrier may be a risk factor for inadequate PCU. We aimed to assess the association between this barrier and inadequate PCU among migrant women.

**Methods:**

This analysis took place in the French multicentre prospective PreCARE cohort study, conducted in four university hospital maternity units in the northern Paris area. It included 10 419 women giving birth between 2010 and 2012. Migrants’ language barrier to communication in French were categorized into three groups: migrants with no, partial or total language barrier. Inadequate PCU was assessed by the date prenatal care began, the proportion of recommended prenatal visits completed and ultrasound scans performed. The associations between these language barrier categories and inadequate PCU were tested with multivariable logistic regression models.

**Results:**

Among the 4803 migrant women included, the language barrier was partial for 785 (16.3%) and total for 181 (3.8%). Compared to migrants with no language barrier, those with partial [risk ratio (RR) 1.23, 95% confidence interval (CI) 1.13–1.33] and total (RR 1.28, 95% CI 1.10–1.50) language barrier were at higher risk of inadequate PCU. Adjustment for maternal age, parity and region of birth did not modify these associations, which were noted particularly among socially deprived women.

**Conclusion:**

Migrant women with language barrier have a higher risk of inadequate PCU than those without. These findings underscore the importance of targeted efforts to bring women with language barrier to prenatal care.

## Introduction

Over the past decade, the migrant population in countries belonging to the Organization for Economic Co-operation and Development has increased by 23%^1^ and that in European Union members by 28%.^2^ Women accounted for 51% of these migrants, and most of them were of childbearing age. As a result, every fourth birth in Western Europe is to a foreign-born mother.^3^^,^^4^ In these host countries, health disparities for outcomes such as maternal mortality^5^ and severe maternal morbidity^6–8^ have been observed between migrant and native women; both are twice as high for migrants than for women born in the host country.

The mechanisms explaining these social health inequalities remain unclear. One likely causal pathway is inadequate prenatal care; it has been reported to be more frequent among migrant than native women in various settings.^9–12^ As language barriers may impair women’s ability to interact with the health system and obtain quality care, they may be a potentially modifiable risk factor for inadequate prenatal care utilization (PCU).

Few studies have explored the specific association of language barrier with inadequate PCU. The very limited available literature has thus far focused mainly on qualitative analyses of the difficulties of health care access for migrants with such barrier.^13^^,^^14^ Exploring if and how language barrier may be associated with inadequate PCU could provide insights into the causal mechanisms of health disparities among migrant women and into the possibilities for preventive interventions. The French multicentre prospective PreCARE cohort, as one of the few databases including information on this barrier among migrant pregnant women, offers the opportunity to explore this issue. Thus, our aim was to assess the association between language barrier and inadequate PCU among migrant women.

## Methods

### Population

**Figure 1:**
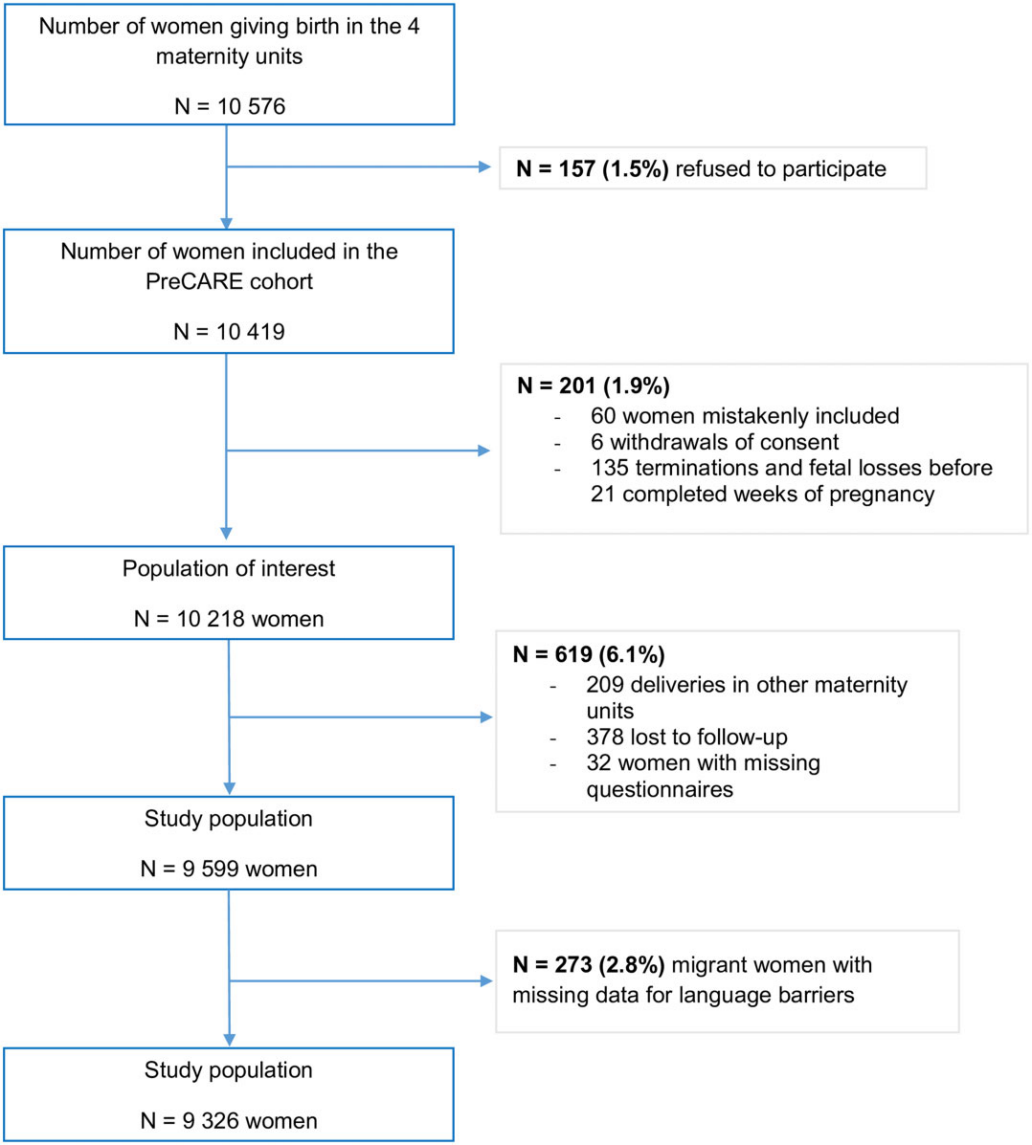
Study population selection

The French PreCARE multicentre prospective cohort study took place in four university hospital maternity units in the northern Paris area from September 2010 to May 2012.^10^^,^^12^ All four maternity hospitals had prenatal care procedures that met the recommendations of the French National Health Authority.^15^ They also worked with the same professional interpreting service which operates throughout France and covers more than 185 languages. Although in theory available 24 hours a day, in the four PreCARE maternity units, this service was mainly used by telephone for scheduled care, in particular antenatal consultations, and had to be anticipated by scheduling a translation shift for rare languages. The study included all pregnant women ≥18 years old, registered and giving birth at these hospitals. This analysis covered the study population of women who gave birth after 21 completed weeks of gestation. It excluded women who finally gave birth in a different maternity units were lost to follow-up, or had missing questionnaires. Migrant women with missing data for language barrier were also excluded (*N* = 273; 2.8%) (figure 1). These women had similar characteristics as those included in the study population (Supplementary table S1).

The regional ethics review board, CPP-Ile-de-France III (No. 09.341bis, 19 November 2009), and the CNIL (Commission Nationale Informatique et Liberté) approved this study. Each woman provided oral informed consent, in accordance with French law. Women were not involved in the development of the research.

### Data collection

Data on social and demographic characteristics (age, deprivation index, education level, social welfare coverage at inclusion, maternal region of birth, length of residency and legal status) were collected by self-administered questionnaires at inclusion and repeated during the postpartum period before discharge. To enable the inclusion of women not speaking French fluently or who could not read or write, these questionnaires were available in English, Chinese and Romanian, and a research assistant or interpreter helped in their completion when needed. Data on women’s medical history and information about their pregnancy, labour, delivery and postpartum period were collected by research assistants and practitioners (midwives and obstetricians) with specific questionnaires completed from the medical files in the postpartum period before discharge.

### Definition of language barrier

The exposure of interest, the migrants’ language barrier to French communication, was categorized into three groups: (i) no language barrier, (ii) a partial language barrier and (iii) a total language barrier. It was assessed by the research assistant at inclusion and was evaluated according to the woman’s ability to read and answer the study’s questionnaire in French and to interact with the research assistant in French. Research assistants at the different inclusion sites received the same training on how to define language barrier to homogenize language barrier classification and minimize bias.

### Definition of inadequate prenatal care utilization

The main outcome was inadequate PCU, categorized as a binary variable. PCU was assessed with three components: (i) late initiation of prenatal care (>14 weeks of gestation), (ii) proportion of prenatal visits completed of the number recommended according to gestational age at delivery (extra visits to check maternal blood pressure or for foetal heart monitoring were not counted in the number of visits) and (iii) the absence of the recommended ultrasound scans in the first (at 11–14 weeks), second (21–24 weeks) and third trimesters (31–34 weeks). These three components were integrated into a PCU index adapted according to the French prenatal care guidelines from the Adequacy of Prenatal Care Utilization (APNCU) index.^16^^,^^17^ The precise method of calculating this modified APNCU (mAPNCU) has been described in a previous publication.^12^ In mAPNCU-1, PCU was considered inadequate if care did not begin until 14 completed weeks of gestation, or if the percentage of prenatal visits was <50% of the recommended number. The mAPNCU-2 further incorporated the recommended ultrasounds: some women with missing ultrasounds were reclassified to the inadequate category (if the first-trimester ultrasound or both of the latter ultrasounds were missing). Other items (preanesthesia evaluation ≥37 weeks, no determination of blood group before entering the delivery room and no hepatitis B serology determination before entering the delivery room) that may be indirect indicators of suboptimal prenatal care were also reported.

### Definition of covariables

Maternal social deprivation was synthesized at the beginning of pregnancy by a previously described^10^ quantitative deprivation index that was the sum of four dimensions: social isolation, poor or insecure housing conditions, no standard health care insurance, and no work-related household income.

Women’s legal status was categorized into four groups: (i) non-migrants, i.e. women born in France, (ii) legal migrants with French or other European Union-27 citizenship, (iii) other legal migrants, with non-European citizenship and (iv) undocumented migrants. The precise definition of the women’s legal status has been described in a previous publication.^18^

A high-risk pregnancy was defined in accordance with French guidelines by the presence of at least one of the following conditions at the beginning of pregnancy: history of cardiac disease, hypertension, diabetes, venous thrombosis, pulmonary embolism, Graves’ disease, asthma, homozygous sickle cell disease, anaemia, thrombocytopenia, coagulation disorder, systemic disease, nephropathy, HIV infection, and a history of late miscarriage, preeclampsia, foetal growth restriction, preterm delivery and foetal or neonatal death.^15^

### Statistical analysis

We calculated the prevalence of language barrier among migrant women. We described women’s baseline characteristics and rates of inadequate PCU by migrant status and language barrier category, expressing qualitative variables as percentages and quantitative variables as their medians and interquartile ranges. In these descriptive analyses, the exposure of interest was a categorical variable in four groups: (i) natives, (ii) migrants with no language barrier, (iii) migrants with a partial language barrier and (iv) migrants with a total language barrier.

We used logistic regression models to assess the association between migrants’ language barrier and inadequate PCU. In these analyses, the exposure of interest was categorized into three groups: (i) migrants with no language barrier, as the reference group; (ii) migrants with a partial language barrier and (iii) migrants with a total language barrier. We used a directed acyclic graph to represent causal assumptions between migrants’ language barrier, inadequate PCU and covariates and to depict the exposure–outcome relations with confounding and intermediate factors. This graph helped to select variables that are potential confounders (i.e. variables associated with both the exposure, which is the migrants’ language barrier, and the outcome of inadequate PCU) from those on the causal pathway between the migrants’ language barrier and inadequate PCU (intermediate factors).^19^ In particular, social vulnerability measured by a deprivation index was considered a potentially intermediate factor, and not a confounder, and thus not included in the multivariable model. The adjustment was performed in successive steps. The first model included demographic variables such as maternal age and parity (in three classes: 0, 1 or ≥2). The second model included the same variables as the first model and the mothers' regions of birth [in six classes: Europe (other than France), North Africa, Sub-Saharan Africa, Asia, Middle East and Other]. The third model included the same variables as the second model and recent immigration (i.e. arrived in France less than 12 months before conception). The fourth model included the same variables as the third model and education level (in four classes: ≤primary school, middle school, high school, and post-secondary schooling). The linearity of the association of the continuous variables (maternal age and number of previous pregnancies) with inadequate PCU was tested. The collinearity between maternal region of birth and recent immigration was also tested.

The proportion of women with missing data in the final multivariable model was 21.0%. Multiple imputation using chained equations (20 datasets) was performed to handle the missing data, assumed to be missing at random (Supplementary table S2).^20^ The results are presented with imputed data as adjusted risk ratios with their 95% confidence intervals (95% CIs). Analyses were performed with STATA software, version 13.1 (Stata Corporation, College Station, TX, USA).

## Results

Among the 10 576 women asked to participate in the PreCare study, 10 419 agreed (98.5%). After the exclusion of women mistakenly included (*n* = 60), or who withdrew their consent (*n* = 6), gave birth before 21 completed weeks of gestation (*n* = 135) or in a non-participating maternity unit (*n* = 209), were lost to follow-up (*n* = 378), or had missing questionnaires (*n* = 32), or missing data for a language barrier (*n* = 273), the analysis included 9326 women (figure 1).

In the study population, 4523 women were born in France (48.5%), 3837 were migrants with no language barrier (41.1%), 785 were migrants with a partial language barrier (8.4%) and 181 women were migrants with a total language barrier (2.0%). One in five migrant women (20.1%) had a language barrier (table 1). Table 1 summarizes the women's baseline characteristics according to their migrant status and language barrier. Migrants with any language barrier had lived less time in France and were more frequently socially isolated, with poor housing conditions, no standard health care insurance, and no work-related household income than were either natives or migrants with no language barrier. Fifty-eight percent of migrants with any language barrier had at least one criterion of maternal social deprivation compared to 19% of natives and 43% of migrants with no language barrier. One-third of migrants with any language barrier had not completed primary school, compared with 9% among those without any language barrier. Compared with migrants with a partial language barrier, those with a total language barrier were more frequently covered by state medical assistance or had no health insurance, were mainly born in Asia, had resided for less time in France, were younger and less frequently at high medical risk at the beginning of their pregnancy (table 1).

**Table 1:** Women’s baseline characteristics by migrant status and language barriers (*N* = 9326)

	Natives	Migrants		
	(*n* = 4523)	No language barrier (*n* = 3837)	Partial language barriers (*n* = 785)	Total language barriers (*n* = 181)
**% Among all women (*N* = 9326)**	48.5	41.1	8.4	2.0
**% Among migrant women (*N* = 4803)**	–	79.9	16.3	3.8
	*n* (%)	*n* (%)	*n* (%)	*n* (%)
**Age (years)**				
<20	76 (1.7)	27 (0.7)	10 (1.3)	8 (4.4)
20–25	606 (13.4)	493 (12.8)	131 (16.7)	36 (19.9)
25–30	1428 (31.6)	1089 (28.4)	250 (31.8)	55 (30.4)
30–35	1553 (34.3)	1185 (30.9)	237 (30.2)	50 (27.6)
35–40	702 (15.5)	812 (21.2)	107 (13.6)	24 (13.3)
≥40	158 (3.5)	231 (6.0)	50 (6.4)	8 (4.4)
Social isolation	77 (1.7)	283 (7.4)	47 (6.0)	5 (2.8)
Poor or insecure housing condition	412 (9.1)	779 (20.3)	209 (26.6)	57 (31.5)
No standard health care insurance	396 (8.8)	1207 (31.5)	350 (44.6)	112 (61.9)
No work-related household income	435 (9.6)	758 (19.8)	190 (24.2)	51 (28.2)
**Deprivation index^a^**				
0 criterion	3661 (80.9)	2133 (55.6)	325 (41.4)	54 (29.8)
1 criterion	515 (11.4)	820 (21.4)	224 (28.5)	60 (33.1)
≥2 criteria	343 (7.6)	827 (21.6)	216 (27.6)	62 (34.2)
**Education level**				
≤Primary school	32 (0.7)	329 (8.6)	222 (28.3)	54 (29.8)
Middle school	643 (14.2)	795 (20.7)	206 (26.2)	43 (23.8)
High school	892 (19.7)	1088 (28.4)	189 (24.1)	45 (24.9)
Post-secondary	2943 (65.1)	1569 (40.9)	156 (19.9)	34 (18.8)
**Social welfare coverage at inclusion**				
SHI + complementary health insurance	3695 (81.7)	1920 (50.0)	225 (28.7)	29 (16.0)
Standard health insurance (SHI)	429 (9.5)	657 (17.1)	191 (24.3)	35 (19.3)
Universal health coverage (CMU)	366 (8.1)	635 (16.5)	120 (15.3)	28 (15.5)
State medical assistance (AME)	1 (0.0)	354 (9.2)	158 (20.1)	48 (26.5)
No healthcare insurance	29 (0.6)	218 (5.7)	72 (9.2)	36 (19.9)
**Maternal region of birth**				
France	4523 (100)	0 (0.0)	0 (0.0)	0 (0.0)
Europe (others)	0 (0.0)	332 (8.7)	79 (10.1)	38 (21.0)
North Africa	0 (0.0)	1662 (43.3)	281 (35.8)	37 (20.4)
Sub-Saharan Africa	0 (0.0)	1268 (33.0)	203 (25.9)	23 (12.7)
Asia	0 (0.0)	299 (7.8)	159 (20.3)	69 (38.1)
Middle East	0 (0.0)	51 (1.3)	19 (2.4)	6 (3.3)
Other	0 (0.0)	225 (5.9)	44 (5.6)	8 (4.4)
Length of residency (median in month)^b^	NA	96.8	46.6	19.3
IQR 25/75	NA	43.1/158	12.4/92.8	6.2/63.2
Smoker before pregnancy	1237 (27.3)	333 (8.7)	31 (3.9)	10 (5.5)
Smoker during pregnancy	644 (14.2)	159 (4.1)	20 (2.5)	8 (4.4)
Alcohol during pregnancy	96 (2.1)	100 (2.6)	11 (1.4)	2 (1.1)
Drugs during pregnancy	39 (0.9)	12 (0.3)	0 (0.0)	0 (0.0)
**Legal status**				
Non-migrants	4523 (100.0)	0 (0.0)	0 (0.0)	0 (0.0)
Legal migrants with French or European citizenship	0 (0.0)	1320 (34.4)	124 (15.8)	26 (14.4)
Other legal migrants	0 (0.0)	2072 (54.0)	480 (61.1)	95 (52.5)
Undocumented migrants	0 (0.0)	445 (11.6)	181 (23.1)	60 (33.1)
**Obstetric history**				
Nulliparous	2302 (50.9)	1342 (35.0)	284 (36.2)	73 (40.3)
Previous caesarean section	178 (3.9)	310 (8.1)	75 (9.6)	10 (5.5)
Voluntary abortion	974 (21.5)	816 (21.3)	85 (10.8)	15 (8.3)
Ectopic pregnancy	90 (2.0)	77 (2.0)	13 (1.7)	2 (1.1)
Late miscarriage	49 (1.1)	78 (2.0)	11 (1.4)	1 (0.6)
Gestational diabetes	136 (3.0)	169 (4.4)	41 (5.2)	6 (3.3)
Pregnancy related hypertensive disorder	99 (2.2)	112 (2.9)	22 (2.8)	3 (1.7)
Foetal growth restriction	62 (1.4)	63 (1.6)	13 (1.7)	1 (0.6)
Preterm delivery	218 (4.8)	221 (5.8)	64 (8.2)	5 (2.8)
Postpartum haemorrhage	59 (1.3)	85 (2.2)	18 (2.3)	3 (1.7)
Foetal or neonatal death	100 (2.2)	108 (2.8)	30 (3.8)	6 (3.3)
High risk at the beginning of pregnancy^c^	925 (20.5)	743 (19.4)	144 (18.3)	19 (10.5)

IQR, interquartile range; NA, not applicable.

a Deprivation index: simple sum of four deprivation dimensions: social isolation, poor or insecure housing condition, no work-related household income, and no permanent health care insurance.

b Among migrants.

c High-risk pregnancy is defined by at least one of the following items in accordance with French guidelines: history of cardiac disease, hypertension, diabetes, venous thrombosis, pulmonary embolism, Graves’ disease, asthma, homozygous sickle cell, anaemia, thrombocytopaenia, coagulation disorder, a rare or systemic disease, nephropathy, HIV infection, and a history of late miscarriage, pre-eclampsia, foetal growth restriction, preterm delivery and foetal or neonatal death.

Migrant women with a language barrier, especially those with a total language barrier, had a higher frequency of inadequate PCU compared with both natives and migrants with no language barrier (table 2). Compared with natives and migrants with no language barrier, migrants with any language barrier had more frequently had their preanesthesia evaluation after 37 gestational weeks and had not had their blood or and hepatitis B serology determined before entering the delivery room (table 2).

**Table 2:** Inadequate prenatal care utilization by migrant status and language barriers

	Natives	Migrants		
	(*n* = 4523)	No language barrier (*n* = 3837)	Partial language barriers (*n* = 785)	Total language barriers (*n* = 181)
	*n* (%)	*n* (%)	*n* (%)	*n* (%)
Initiation of care ≥14 GW (*N* = 9316)	641 (14.2)	713 (18.6)	185 (23.6)	45 (24.9)
Percentage of recommended prenatal visits <50%^a^ (*N* = 9297)	118 (2.6)	101 (2.6)	38 (4.8)	11 (6.1)
First-trimester ultrasound not performed between 11 and 14 GW (*N* = 8999)	623 (13.8)	929 (24.2)	263 (33.5)	61 (33.7)
Second trimester ultrasound not performed between 21 and 24 GW (*N* = 9134)	594 (13.1)	725 (18.9)	178 (22.7)	58 (32.0)
Third trimester ultrasound not performed between 31 and 34 GW (*N* = 9173)	679 (15.0)	669 (17.4)	155 (19.7)	50 (27.6)
**Inadequate prenatal care according to mAPNCU-1 index^b^**	723 (16.0)	772 (20.1)	198 (25.2)	49 (27.1)
Missing data for an item of the index	9 (0.2)	12 (0.3)	1 (0.1)	3 (1.7)
**Inadequate prenatal care according to mAPNCU-2 index^c^**	1196 (26.4)	1448 (37.7)	363 (46.2)	86 (47.5)
Missing data for an item of the index	307 (6.8)	249 (6.5)	46 (5.9)	10 (5.5)
**Indirect indicators of prenatal care**				
Preanesthesia evaluation ≥37 GW (*N* = 8815)	438 (9.7)	433 (11.3)	102 (13.0)	28 (15.5)
No determination of blood group before entering the delivery room (*N* = 9263)	23 (0.5)	16 (0.4)	8 (1.0)	2 (1.1)
No hepatitis B serology determination before entering the delivery room (*N* = 9281)	33 (0.7)	18 (0.5)	4 (0.5)	2 (1.1)

Percentages are calculated in columns. GW, gestation weeks.

a Percentage of recommended prenatal visits according to pregnancy duration.

b mAPNCU-1 index, which considers initiation of care and percentage of recommended prenatal visits made.

c mAPNCU-2 index, which considers initiation of care, percentage of recommended prenatal visits made, and recommended ultrasound scans performed.

In the univariate analysis, compared with migrants with no language barrier, migrants with a total language barrier had a higher risk of inadequate PCU for both the mAPNCU-1 [27% vs. 20%, risk ratio (RR) 1.38; 95% CI 1.08–1.76] and the mAPNCU-2 index (48% vs. 38%, RR 1.28; 95% CI 1.10–1.50); migrants with partial language barrier also had a higher risk of inadequate PCU for both the mAPNCU-1 (25% vs. 20%, RR 1.25; 95% CI 1.09–1.43) and the mAPNCU-2 index (46% vs. 38%, RR 1.23; 95% CI 1.13–1.33) (table 3).

**Table 3:** Risk of inadequate prenatal care utilization among migrant women by language barriers (multiple imputation)

	Inadequate prenatal care—mAPNCU-1 index^a^				
	RR [95% CI]	Model 1	Model 2	Model 3	Model 4
		aRR [95% CI]	aRR [95% CI]	aRR [95% CI]	aRR [95% CI]
Migrants with no language barrier	1	1	1	1	1
Migrants with partial language barriers	1.25 [1.09–1.43]	1.21 [1.06–1.39]	1.23 [1.08–1.42]	1.14 [0.99–1.31]	1.06 [0.92–1.22]
Migrants with total language barriers	1.38 [1.08–1.76]	1.33 [1.04–1.69]	1.41 [1.09–1.81]	1.19 [0.93–1.54]	1.10 [0.85–1.42]

	Inadequate prenatal care—mAPNCU-2 index^b^				
	RR [95% CI]	Model 1	Model 2	Model 3	Model 4
		aRR [95% CI]	aRR [95% CI]	aRR [95% CI]	aRR [95% CI]
Migrants with no language barrier	1	1	1	1	1
Migrants with partial language barriers	1.23 [1.13–1.33]	1.20 [1.10–1.30]	1.23 [1.13–1.34]	1.16 [1.06–1.26]	1.09 [1.00–1.19]
Migrants with total language barriers	1.28 [1.10–1.50]	1.25 [1.07–1.45]	1.36 [1.16–1.60]	1.19 [1.01–1.39]	1.11 [0.95–1.31]

Multiple imputation using chained equations (20 datasets) was performed. aRR, adjusted risk ratio; CI, confidence interval; RR, risk ratio.

Multivariable models among 4803 migrant women after multiple imputation:

Model 1 adjusted for maternal age and parity (in three classes: 0, 1 or ≥2).

Model 2 adjusted for same variables as Model 1 and maternal region of birth [in six classes: Europe (others than France), North Africa, Sub-Saharan Africa, Asia, Middle East and Other].

Model 3 adjusted for same variables as Model 2 and recent immigration (i.e. arrived in France less than 12 months before conception).

Model 4 adjusted for same variables as Model 3 and education level (in four classes: ≤primary school, middle school, high school or post-secondary).

a Based on the mAPNCU-1 index (modified Adequacy of Prenatal Care Utilization index), which considers initiation of care, and percentage of recommended prenatal visits made.

b Based on the mAPNCU-2 index (modified Adequacy of Prenatal Care Utilization index), which considers initiation of care, percentage of recommended prenatal visits made and ultrasound scans performed.

In the multivariable analysis, these associations between language barrier and inadequate PCU for both the mAPNCU-1 and mAPNCU-2 index did not change when adjusted for maternal age, parity, and region of birth (Model 2, table 3). They were weaker after further adjustment for recent immigration (Model 3, table 3) and education level (Model 4, table 3). In stratified analyses according to the deprivation index, associations between language barrier and inadequate PCU were significant for both indices in socially deprived women (Supplementary table S3).

Supplementary table S4 shows the crude and adjusted RRs for inadequate PCU for the covariables included in Model 4 of table 3.

Results were similar in the complete case analysis (Supplementary table S5).

## Discussion

### Main findings

Migrants with a language barrier, whether total or partial, had a higher risk of inadequate PCU than migrants without a language barrier. They also had resided in France for less time and were more frequently socially deprived and undocumented than the migrants with no language barrier.

### Strengths and limitations

To our knowledge, this study is one of the very few based on prospective multicentre data able to explore the association between language barrier and inadequate PCU. A strength of this study is its geographic setting—an area chosen to allow us to recruit a multicultural cohort consistent with our scientific objectives. The large sample of migrant women with language barrier provides adequate statistical power, even though it produces a population not representative of that of France. However, no population-based studies in France, including the French national perinatal surveys, have collected data on language barrier.^21^ The data collection method, especially the availability of the questionnaires in four different languages and the availability of a research assistant or interpreter to help complete it, enabled us to include migrant women with language barrier and reduce both the risk of selection bias and the missing data rate. Another strength is that we were able to assess specifically the association of the language barrier with inadequate PCU, by adjusting for confounding factors that may have affected PCU, particularly the length of residency and maternal education. Nevertheless, the substantial number of migrant women with missing data for at least one variable included in the final multivariate analysis (21.0%) remains a limitation. However, the comparisons of results obtained by the analyses with imputed data and with complete cases show that these had a very limited impact on the results. Although 2.8% of women could not be included in the study population because of missing information on language barrier, this percentage remains small and is unlikely to bias our results as the characteristics of these women did not differ from those of the women analysed (Supplementary table S1). The categorization of language barrier was not based on specific language tests and could not take into account the more subtle variations in language understanding, speaking and writing among women with partial barrier. An interpreting service was available in all four maternity units, limiting the external validity. In maternity hospitals without professional interpreting services, we would thus have expected the strength of the association between language barrier and inadequate PCU to be stronger. However, we are not able to document how the interpreting services were used for each woman in the participating maternity units. The recruitment period of the PreCARE cohort may limit the generalizability of our findings to the current context. However, the organization of the health care system and the profile of migrant to France has remained quite similar over the past decade.^22^ Finally, our definition of inadequate PCU is purely quantitative and does not reflect its qualitative content. It nonetheless incorporates key elements of prenatal care.

### Interpretation

Our analysis shows that compared with migrants with no language barrier, migrants with language barrier, either total or partial, had a higher risk of inadequate PCU.

We found that migrants with language barrier had resided in France for less time and were more frequently socially deprived and undocumented than those without. It highlights that a language barrier is a marker of a subgroup of migrant women at risk and should be considered a warning signal for clinicians and hospital’s administrative staff, particularly with regard to their PCU.

Language barrier may also directly impair women’s ability to interact with the health system and obtain quality care, as well as with the administration to access their rights, particularly for those who immigrated recently, as our results underline.^10^ Beyond communication difficulties, language barrier may also activate discriminatory attitudes leading to differential care.^7^^,^^23^ To further explore these mechanisms, qualitative methods would be a valuable complement to our quantitative analysis.

Interestingly, although women born in sub-Saharan Africa have a higher risk of inadequate PCU^10^ and adverse maternal outcomes, ^24–30^ the higher risk of inadequate PCU for migrants with language barrier did not change after adjustment for maternal region of birth. This suggests that this barrier does not explain the risk profile of migrant women from sub-Saharan Africa in France.

By assessing the association between language barrier and inadequate PCU, we explored a small part of the whole health literacy skills to access, understand, apply health information and take an active part in decisions that affect their health. It would have been interesting to integrate other dimensions of health literacy. However, these were not collected in the PreCARE cohort because of practical feasibility. An ancillary socio-anthropological study of the PreCARE cohort was conducted through interviews with women and includes dimensions of health literacy; its analysis is ongoing.^31^

## Conclusion

In maternity units using interpreting services, migrant women with language barrier, either total or partial, have a higher risk of inadequate PCU than those with no such barrier. Regardless of whether the language barrier is only a marker of a high-risk subgroup or also a causal factor, these findings underscore the importance of targeted efforts from health care providers and also from administrative staff, to bring women with language barrier to prenatal care.

## Supplementary Material

ckad078_Supplementary_Data

## Data Availability

The data underlying this article are available in the article and in its supplementary material.
