# Supplementary material for: Association between language barrier and inadequate prenatal care utilization among migrant women in the PreCARE prospective cohort study
Source: Eur J Public Health. 2023 May 16;33(3):403–10. doi: 10.1093/eurpub/ckad078 (PMC10234658; doi:10.1093/eurpub/ckad078)
Supplement: ckad078_Supplementary_Data [file ckad078_supplementary_data.docx]

Table S1. Comparison of the characteristics of migrant women included in the study population and those with missing data for language barrier.

|  | | **Migrant women included in the study population**  **(n = 4803)** | | **Migrant women with missing data for language barrier**  **(n = 273)** | |
| --- | --- | --- | --- | --- | --- |
|  | | n | % | n | % |
| **Age (years)** | |  |  |  |  |
|  | < 20 | 45 | 0.9 | 7 | 2.6 |
|  | [20 – 25[ | 660 | 13.7 | 37 | 13.6 |
|  | [25 – 30[ | 1394 | 29.0 | 90 | 33.0 |
|  | [30 – 35[ | 1472 | 30.6 | 83 | 30.4 |
|  | [35 – 40[ | 943 | 19.6 | 40 | 14.7 |
|  | ≥ 40 | 289 | 6.0 | 16 | 5.9 |
| Social isolation | | 335 | 7.0 | 15 | 5.5 |
| Poor or insecure housing condition | | 1045 | 21.8 | 46 | 16.8 |
| No standard health care insurance | | 1669 | 34.7 | 79 | 28.9 |
| No work-related household income | | 999 | 20.8 | 50 | 18.3 |
| **Deprivation index *** | |  |  |  |  |
|  | 0 criterion | 2512 | 52.3 | 153 | 56.0 |
|  | 1 criterion | 1104 | 23.0 | 62 | 22.7 |
|  | ≥ 2 criteria | 1105 | 23.0 | 51 | 18.7 |
| **Education level** | |  |  |  |  |
|  | ≤ Primary school | 605 | 12.6 | 30 | 11.0 |
|  | Middle school | 1044 | 21.7 | 59 | 21.6 |
|  | High school | 1322 | 27.5 | 85 | 31.1 |
|  | Post-secondary | 1759 | 36.6 | 90 | 33.0 |
| **Social welfare coverage at inclusion** | |  |  |  |  |
|  | Standard health insurance (SHI) | 883 | 18.4 | 56 | 20.5 |
|  | SHI + Complementary health insurance | 2174 | 45.3 | 132 | 48.4 |
|  | Universal health coverage (CMU) | 783 | 16.3 | 42 | 15.4 |
|  | State medical assistance (AME) | 560 | 11.7 | 25 | 9.2 |
|  | No healthcare insurance | 326 | 6.8 | 12 | 4.4 |
| **Maternal birthplace** | |  |  |  |  |
|  | Europe (others) | 449 | 9.3 | 18 | 6.6 |
|  | North Africa | 1980 | 41.2 | 139 | 50.9 |
|  | Sub-Saharan Africa | 1494 | 31.1 | 75 | 27.5 |
|  | Middle East | 76 | 1.6 | 2 | 0.7 |
|  | Asia | 527 | 11.0 | 21 | 7.7 |
|  | Other | 277 | 5.8 | 18 | 6.6 |
| Length of residency (median in month)  IQR 25/75 | | 83.0  31.2/141.4 | | 91.5  31.9/157.1 | |
| Smoker before pregnancy | | 374 | 7.8 | 15 | 5.5 |
| Smoker during pregnancy | | 187 | 3.9 | 13 | 4.8 |
| Alcohol during pregnancy | | 113 | 2.4 | 2 | 0.7 |
| Drugs during pregnancy | | 12 | 0.2 | 0 | 0.0 |
| **Legal Status** | |  |  |  |  |
|  | Legal migrants with French or European citizenship | 1470 | 30.6 | 85 | 31.1 |
|  | Other legal migrants | 2647 | 55.1 | 159 | 58.2 |
|  | Undocumented migrants | 686 | 14.3 | 29 | 10.6 |
| **Obstetric history** | |  |  |  |  |
|  | Nulliparous | 1699 | 35.4 | 112 | 41.0 |
|  | Previous caesarean delivery | 395 | 8.2 | 18 | 6.6 |
|  | Voluntary abortion | 916 | 19.1 | 34 | 12.5 |
|  | Ectopic pregnancy | 92 | 1.9 | 5 | 1.8 |
|  | Late miscarriage | 90 | 1.9 | 0 | 0.0 |
|  | Gestational diabetes | 216 | 4.5 | 16 | 5.9 |
|  | Pregnancy related hypertensive disorder | 137 | 2.9 | 4 | 1.5 |
|  | Fetal growth restriction | 77 | 1.6 | 3 | 1.1 |
|  | Preterm delivery | 290 | 6.0 | 13 | 4.8 |
|  | Postpartum haemorrhage | 106 | 2.2 | 4 | 1.5 |
|  | Fetal or neonatal death | 144 | 3.0 | 5 | 1.8 |
| High risk at the beginning of pregnancy** | | 906 | 18.9 | 36 | 13.2 |

IQR, interquartile range; NA, not applicable; the sum is not equal to 100% due to missing data

* Deprivation index: simple sum of 4 deprivation dimensions: Social isolation, Poor or insecure housing condition, No work-related household income, and No permanent heath care insurance

** High-risk pregnancy is defined by at least one of the following items in accordance with French guidelines: history of cardiac disease, hypertension, diabetes, venous thrombosis, pulmonary embolism, Graves’ disease, asthma, homozygous sickle cell, anaemia, thrombocytopaenia, coagulation disorder, a rare or systemic disease, nephropathy, HIV infection, and a history of late miscarriage, pre-eclampsia, fetal growth restriction, preterm delivery, and fetal or neonatal death

Table S2. Comparison of the characteristics of migrant women without missing data for any variable included in the multivariate analysis and those with missing data for at least one variable included in the multivariate analysis.

|  | | **Migrant women without missing data for any variable included in the multivariate analysis**  **(n = 3792)** | | **Migrant women with missing data for at least one variable included in the multivariate analysis**  **(n = 1011)** | |
| --- | --- | --- | --- | --- | --- |
|  | | n | % | n | % |
| **Age (years)** | |  |  |  |  |
|  | < 20 | 40 | 1.1 | 5 | 0.5 |
|  | [20 – 25[ | 520 | 13.7 | 140 | 13.8 |
|  | [25 – 30[ | 1129 | 29.8 | 265 | 26.2 |
|  | [30 – 35[ | 1157 | 30.5 | 315 | 31.2 |
|  | [35 – 40[ | 726 | 19.1 | 217 | 21.5 |
|  | ≥ 40 | 220 | 5.8 | 69 | 6.8 |
| Social isolation | | 251 | 6.6 | 84 | 8.3 |
| Poor or insecure housing condition | | 823 | 21.7 | 222 | 22.0 |
| No standard health care insurance | | 1321 | 34.8 | 348 | 34.4 |
| No work-related household income | | 785 | 20.7 | 214 | 21.2 |
| **Deprivation index *** | |  |  |  |  |
|  | 0 criterion | 1990 | 52.5 | 522 | 51.6 |
|  | 1 criterion | 872 | 23.0 | 232 | 22.9 |
|  | ≥ 2 criteria | 871 | 23.0 | 234 | 23.1 |
| **Education level** | |  |  |  |  |
|  | ≤ Primary school | 480 | 12.7 | 125 | 12.4 |
|  | Middle school | 820 | 21.6 | 224 | 22.2 |
|  | High school | 1070 | 28.2 | 252 | 24.9 |
|  | Post-secondary | 1422 | 37.5 | 337 | 33.3 |
| **Social welfare coverage at inclusion** | |  |  |  |  |
|  | Standard health insurance (SHI) | 696 | 18.4 | 187 | 18.5 |
|  | SHI + Complementary health insurance | 1718 | 45.3 | 456 | 45.1 |
|  | Universal health coverage (CMU) | 619 | 16.3 | 164 | 16.2 |
|  | State medical assistance (AME) | 448 | 11.8 | 112 | 11.1 |
|  | No healthcare insurance | 254 | 6.7 | 72 | 7.1 |
| **Maternal birthplace** | |  |  |  |  |
|  | Europe (others) | 346 | 9.1 | 103 | 10.2 |
|  | North Africa | 1614 | 42.6 | 366 | 36.2 |
|  | Sub-Saharan Africa | 1157 | 30.5 | 337 | 33.3 |
|  | Middle East | 56 | 1.5 | 20 | 2.0 |
|  | Asia | 419 | 11.0 | 108 | 10.7 |
|  | Other | 200 | 5.3 | 77 | 7.6 |
| Length of residency (median in month)  IQR 25/75 | | 82.7  31.2/140.6 | | 89.0  33.6/155.9 | |
| Smoker before pregnancy | | 277 | 7.3 | 97 | 9.6 |
| Smoker during pregnancy | | 131 | 3.5 | 56 | 5.5 |
| Alcohol during pregnancy | | 85 | 2.2 | 28 | 2.8 |
| Drugs during pregnancy | | 10 | 0.3 | 2 | 0.2 |
| **Legal Status** | |  |  |  |  |
|  | Legal migrants with French or European citizenship | 1117 | 29.5 | 353 | 34.9 |
|  | Other legal migrants | 2134 | 56.3 | 513 | 50.7 |
|  | Undocumented migrants | 541 | 14.3 | 145 | 14.3 |
| **Obstetric history** | |  |  |  |  |
|  | Nulliparous | 1372 | 36.2 | 327 | 32.3 |
|  | Previous caesarean delivery | 295 | 7.8 | 100 | 9.9 |
|  | Voluntary abortion | 723 | 19.1 | 193 | 19.1 |
|  | Ectopic pregnancy | 73 | 1.9 | 19 | 1.9 |
|  | Late miscarriage | 68 | 1.8 | 22 | 2.2 |
|  | Gestational diabetes | 167 | 4.4 | 49 | 4.8 |
|  | Pregnancy related hypertensive disorder | 105 | 2.8 | 32 | 3.2 |
|  | Fetal growth restriction | 56 | 1.5 | 21 | 2.1 |
|  | Preterm delivery | 220 | 5.8 | 70 | 6.9 |
|  | Postpartum haemorrhage | 86 | 2.3 | 20 | 2.0 |
|  | Fetal or neonatal death | 100 | 2.6 | 44 | 4.4 |
| High risk at the beginning of pregnancy** | | 684 | 18.0 | 222 | 22.0 |

IQR, interquartile range; NA, not applicable; the sum is not equal to 100% due to missing data

* Deprivation index: simple sum of 4 deprivation dimensions: Social isolation, Poor or insecure housing condition, No work-related household income, and No permanent heath care insurance

** High-risk pregnancy is defined by at least one of the following items in accordance with French guidelines: history of cardiac disease, hypertension, diabetes, venous thrombosis, pulmonary embolism, Graves’ disease, asthma, homozygous sickle cell, anaemia, thrombocytopaenia, coagulation disorder, a rare or systemic disease, nephropathy, HIV infection, and a history of late miscarriage, pre-eclampsia, fetal growth restriction, preterm delivery, and fetal or neonatal death

Table S3. Risk of inadequate prenatal care utilization among migrant women by language barrier and stratified by deprivation index (multiple imputation).

|  |  | **Inadequate prenatal care - mAPNCU-1 index*** | |  | **Inadequate prenatal care** - **mAPNCU-2 index **** | |
| --- | --- | --- | --- | --- | --- | --- |
|  |  | RR [95% CI] | aRR [95% CI] |  | RR [95% CI] | aRR [95% CI] |
| **Deprivation index = 0 Criterion** | |  |  |  |  |  |
|  | Migrants with no language barrier | 1 | 1 |  | 1 | 1 |
|  | Migrants with partial language barrier | 1.05 [0.81-1.35] | 0.93 [0.71-1.22] |  | 1.21 [1.04-1.40] | 1.08 [0.92-1.27] |
|  | Migrants with total language barrier | 0.63 [0.30-1.36] | 0.54 [0.24-1.19] |  | 0.93 [0.62-1.39] | 0.83 [0.55-1.25] |
| **Deprivation index ≥1 Criterion** | |  |  |  |  |  |
|  | Migrants with no language barrier | 1 | 1 |  | 1 | 1 |
|  | Migrants with partial language barrier | 1.22 [1.04-1.42] | 1.08 [0.92-1.27] |  | 1.13 [1.03-1.24] | 1.06 [0.95-1.17] |
|  | Migrants with total language barrier | 1.41 [1.10-1.80] | 1.16 [0.89-1.50] |  | 1.21 [1.03-1.41] | 1.07 [0.90-1.27] |

RR, risk ratio; aRR, adjusted risk ratio; CI, Confidence interval.

Multivariable logistic regression models with multiple imputation adjusted for maternal age, parity (in three classes: 0, 1 or ≥2), maternal region of birth (in six classes: Europe (others than France), North Africa, Sub-Saharan Africa, Asia, Middle East and Other), recent immigration (i.e., arrived in France less than 12 months before pregnancy began), and education level (in four classes: ≤primary school, middle school, high school or post-secondary)

* Based on the mAPNCU-1 index (modified Adequacy of Prenatal Care Utilization index), which considers initiation of care, and percentage of recommended prenatal visits made.

** Based on the mAPNCU-2 index (modified Adequacy of Prenatal Care Utilization index), which considers initiation of care, percentage of recommended prenatal visits made, and ultrasound scans performed.

Table S4. Inadequate prenatal care utilization among migrant women by maternal characteristics (multiple imputation).

|  |  | **Inadequate prenatal care - mAPNCU-1 index*** | |  | **Inadequate prenatal care** - **mAPNCU-2 index **** | |
| --- | --- | --- | --- | --- | --- | --- |
|  |  | RR [95% CI] | aRR [95% CI] |  | RR [95% CI] | aRR [95% CI] |
| Language barrier | |  |  |  |  |  |
|  | No | 1 | 1 |  | 1 | 1 |
|  | Partial | 1.25 [1.09-1.43] | 1.06 [0.92-1.22] |  | 1.23 [1.13-1.33] | 1.09 [1.00-1.19] |
|  | Total | 1.38 [1.08-1.76] | 1.10 [0.85-1.42] |  | 1.28 [1.10-1.50] | 1.11 [0.95-1.31] |
| Maternal age (years) | |  |  |  |  |  |
|  | < 20 | 1.07 [0.63-1.80] | 0.89 [0.52-1.54] |  | 1.25 [0.93-1.67] | 1.10 [0.80-1.51] |
|  | [20 – 25[ | 1.23 [1.05-1.44] | 1.22 [1.04-1.43] |  | 1.18 [1.07-1.30] | 1.19 [1.07-1.31] |
|  | [25 – 30[ | 1 | 1 |  | 1 | 1 |
|  | [30 – 35[ | 0.81 [0.70-0.93] | 0.82 [0.71-0.95] |  | 0.90 [0.82-0.98] | 0.91 [0.83-1.00] |
|  | [35 – 40[ | 0.78 [0.67-0.93] | 0.80 [0.67-0.95] |  | 0.86 [0.77-0.96] | 0.85 [0.77-0.95] |
|  | ≥ 40 | 1.07 [0.86-1.34] | 1.07 [0.85-1.34] |  | 1.02 [0.88-1.18] | 0.99 [0.85-1.16] |
| Parity | |  |  |  |  |  |
|  | 0 | 1 | 1 |  | 1 | 1 |
|  | 1 | 1.04 [0.90-1.19] | 1.14 [0.99-1.31] |  | 1.12 [1.02-1.22] | 1.21 [1.11-1.32] |
|  | ≥2 | 1.14 [1.01-1.30] | 1.26 [1.08-1.47] |  | 1.18 [1.09-1.29] | 1.28 [1.16-1.41] |
| Maternal region of birth | |  |  |  |  |  |
|  | Europe (others) | 0.88 [0.70-1.11] | 0.90 [0.72-1.13] |  | 0.87 [0.77-1.00] | 0.89 [0.77-1.02] |
|  | North Africa | 1 | 1 |  | 1 | 1 |
|  | Sub-Saharan Africa | 1.36 [1.20-1.55] | 1.25 [1.10-1.43] |  | 1.18 [1.09-1.28] | 1.10 [1.01-1.19] |
|  | Middle East | 1.26 [0.83-1.90] | 1.29 [0.86-1.94] |  | 0.96 [0.71-1.29] | 0.98 [0.73-1.31] |
|  | Asia | 1.17 [0.98-1.41] | 1.16 [0.96-1.39] |  | 0.95 [0.84-1.08] | 0.95 [0.84-1.07] |
|  | Other | 1.22 [0.97-1.54] | 1.23 [0.97-1.55] |  | 1.05 [0.90-1.22] | 1.05 [0.90-1.22] |
| Education level | |  |  |  |  |  |
|  | ≤ Primary school | 1.70 [1.45-1.99] | 1.44 [1.21-1.70] |  | 1.53 [1.38-1.69] | 1.34 [1.20-1.49] |
|  | Middle school | 1.28 [1.10-1.49] | 1.18 [1.01-1.38] |  | 1.32 [1.20-1.45] | 1.23 [1.12-1.36] |
|  | High school | 1.25 [1.08-1.44] | 1.16 [1.00-1.34] |  | 1.21 [1.10-1.33] | 1.14 [1.03-1.25] |
|  | Post-secondary | 1 | 1 |  | 1 | 1 |
| Recent immigration | | 1.65 [1.44-1.88] | 1.66 [1.44-1.91] |  | 1.51 [1.40-1.64] | 1.55 [1.42-1.70] |

RR, risk ratio; aRR, adjusted risk ratio; CI, Confidence interval.

Multivariable logistic regression models with multiple imputation including all variables listed in the Table; same as Models 4 of Table 3.

* Based on the mAPNCU-1 index (modified Adequacy of Prenatal Care Utilization index), which considers initiation of care, and percentage of recommended prenatal visits made.

** Based on the mAPNCU-2 index (modified Adequacy of Prenatal Care Utilization index), which considers initiation of care, percentage of recommended prenatal visits made, and ultrasound scans performed.

Table S5. Risk of inadequate prenatal care utilization among migrant women by language barrier (complete-case analysis).

|  | **Inadequate prenatal care - mAPNCU-1 index*** | | | | |
| --- | --- | --- | --- | --- | --- |
|  | RR [95% CI] | Model 1  aRR [95% CI] | Model 2  aRR [95% CI] | Model 3  aRR [95% CI] | Model 4  aRR [95% CI] |
| Migrants with no language barrier | 1 | 1 | 1 | 1 | 1 |
| Migrants with partial language barrier | 1.27 [1.09-1.48] | 1.24 [1.07-1.44] | 1.26 [1.09-1.47] | 1.16 [1.00-1.35] | 1.09 [0.93-1.28] |
| Migrants with total language barrier | 1.34 [1.01-1.79] | 1.31 [0.99-1.75] | 1.39 [1.03-1.86] | 1.15 [0.86-1.54] | 1.07 [0.80-1.44] |
|  | | | | | |
|  | **Inadequate prenatal care - mAPNCU-2 index**** | | | | |
|  | RR [95% CI] | Model 1  aRR [95% CI] | Model 2  aRR [95% CI] | Model 3  aRR [95% CI] | Model 4  aRR [95% CI] |
| Migrants with no language barrier | 1 | 1 | 1 | 1 | 1 |
| Migrants with partial language barrier | 1.24 [1.13-1.36] | 1.22 [1.11-1.33] | 1.25 [1.14-1.37] | 1.17 [1.06-1.28] | 1.11 [1.00-1.22] |
| Migrants with total language barrier | 1.26 [1.06-1.50] | 1.23 [1.04-1.47] | 1.33 [1.11-1.60] | 1.15 [0.96-1.38] | 1.08 [0.90-1.30] |

RR, risk ratio; aRR, adjusted risk ratio; CI, Confidence interval.

* Based on the mAPNCU-1 index (modified Adequacy of Prenatal Care Utilization index), which considers initiation of care, and percentage of recommended prenatal visits made (N=4034).

** Based on the mAPNCU-2 index (modified Adequacy of Prenatal Care Utilization index), which considers initiation of care, percentage of recommended prenatal visits made, and ultrasound scans performed (N=3792).

Models 1 adjusted for maternal age and parity (in three classes: 0, 1 or ≥2),

Models 2 adjusted for same variables as Model 1 and maternal region of birth (in six classes: Europe (others than France), North Africa, Sub-Saharan Africa, Asia, Middle East and Other)

Models 3 adjusted for same variables as Model 2 and recent immigration (i.e., arrived in France less than 12 months before conception)

Models 4 adjusted for same variables as Model 3 and education level (in four classes: ≤primary school, middle school, high school or Post-secondary)
